# Pathology, Practical Medicine, and Therapeutics

**Published:** 1838-04

**Authors:** 


*Med. Gazette. February* 24, 1838.

### PATHOLOGY, PRACTICAL MEDICINE, AND THERAPEUTICS.

*Another instance of supernumerary Mammæ.* By W. H. Moore, Esq., Woodbridge.

Eliza Snell, a healthy-looking woman, aged twenty-three, in October last, being in the fifth month of pregnancy with her second child, observed a tumour in the right axilla, as large as a walnut, which gave her no pain, nor sensation of tenderness on pressure. As it did not produce any inconvenience she took no further notice of it until her admission into the poor-house of the Woodbridge Union, where she was placed for delivery; and in January last she shewed it to me. It had not enlarged since her first perceiving it. I found it moveable, not very hard, free from heat, pain, and tenderness, and ordered it to be poulticed. This was done for eight days, but the poultices seemed to create pain, without either softening or diminishing it. She was delivered early in the present month, and to-day my attention was again called to it by the nurse, who stated that there had been a discharge of milk from it. On examination I saw upon its surface eight or ten papillæ, with a minute pore at the apex of each, and an inflamed areola around its base. On pressing the tumour a white fluid issued in five distinct streams, from as many of the papillæ. These were checked on removing the pressure, but could be made to jet forth to a distance of seven or eight inches on resuming it. I collected nearly a teaspoonful of the fluid, which had just the colour, consistence, and sweetish taste of milk. There is a similar tumour in the axilla of the other side, with papillæ also, but I could force no milk from them; this had never been poulticed. There is nothing remarkable in the woman. The breasts are very abundantly supplied with milk. *Lancet. February* 24, 1838.

---

*On the Frequency of the Presence of the Trichocephalus Dispar in the Human Intestines.* By O'B. Bellingham, m.d., Second Surgeon to St. Vincent's Hospital, &c.

[This paper gives a very good account of this worm, which has been much more observed by foreign pathologists than by our countrymen. Dr. Bellingham, however, shews that it is extremely prevalent in this country, or at least in Dublin. The following passages are the most important in Dr. B.'s communication.]

This particular species (the Trichocephalus Dispar) was formerly called trichuris (derived from θριξ, a hair, and ουρα, a tail,) from the erroneous supposition that the capillary portion was the tail of the animal; it is from an inch and a half to two inches long, the capillary part composing about two-thirds of its length, at the extremity of which is its mouth. The male is smaller than the female, and the thicker part of its body is spirally twisted, in the female it is straight or nearly so; the penis of the male is a simple spiculum, contained in a sheath, the orifice of which is somewhat bell-shaped. The opening, which, in the female, serves the purpose both of vagina and anus, is small, and situated close to the termination of the posterior extremity.

The part of the intestinal canal which the trichocephalus dispar most commonly occupies is the cæcum, more particularly the neighbourhood of the ilio-cæcal valve; I have found them however through the whole tract of the colon, in the ileum close to the cæcum, and in the appendix vermiformis. We sometimes find the head of the animal implanted in the mucous membrane, lining the intestine; but this is rare; more commonly they are quite unattached, and when an opening is made into the cæcum, they come out in its fluid contents.

With a view to determine between the contradictory statements of the English and continental pathologists, I examined successively the intestinal canal of twenty-nine individuals who died in St. Vincent's Hospital during the last twelve months, (of whom eleven were males and eighteen females,) and in twenty-six out of the twenty-nine I found a greater or less number of these worms. The ages of these individuals varied much, the youngest being but eight years old, and the oldest upwards of seventy. The diseases which proved fatal to them also varied: some died of injuries, others of acute, and others of chronic diseases. In several instances I found but two or three trichocephali; in others upwards of eighty; the largest number having been found in a boy aged fourteen, who died of dropsy, with disease of heart and kidneys, in whom I counted one hundred and nineteen. In some instances I examined the proportion which the male trichocephali bore to the females; in one individual I found nineteen males and twenty-five females; in another sixty-one males and twenty-four females; in another one male and one female; in another four, all males; and in another six, all females. The three individuals in whom I failed in detecting these worms, were females; one died of scirrhus of the pylorus; and the communication between the stomach and duodenum, was so contracted as not to admit even a probe to pass, hence we may naturally suppose, that if they had previously existed they were starved out; another died of cancer, which commenced close to the orifice of the urethra; and the third died of protracted diarrhœa, with extensive ulceration of the mucous membrane of the cæcum, colon, and lower part of the ileum; she had been taking for some time before her death a combination of sulphas cupri with opium and other medicines, which most likely acted as poisons to these animals, and caused their expulsion. Not one of the twenty-six individuals in whom these worms existed had complained, either before or during the illness which proved fatal, of any symptom which could lead to the suspicion that these worms had been in the least degree prejudicial to their health.

*Dublin Journal of Med. Science. Jan.* 1838.

---

*Enquiry as to whether Laryngismus Stridulus is a Spasmodic or Paralytic Affection.* By WILLIAM GRIFFIN, M.D., Limerick.

[THIS is an elaborate critical enquiry, and also contains several new and interesting observations on the important subject treated of. We have noticed it so fully on more than one occasion, that we can only here extract Dr. Griffin's concluding paragraph, which places his modesty and candour in a favorable light.]

I have offered these few unsatisfactory observations without wishing to attach more importance to them than they merit. I believe both the pathology and treatment of the disease are still very uncertain, and that it will require all the consideration and enquiry which observant practitioners can bestow upon the subject for many future years to attain a just knowledge of either. The following summary of the amount of our present information and of the facts connected with the disease, may be useful to subsequent enquirers:—1. By the concurrent testimony of almost all who have noticed the affection, it occurs for the most part, if not wholly, in strumous habits. 2. It is frequently found in connexion with enlarged glands in the neck, and perhaps in the thorax. 3. It is frequently found in connexion with eruptions on the face, ears, or scalp. 4. It frequently terminates in convulsions, and is sometimes, though very rarely, ushered in by them. I believe it may be said that nearly half the fatal cases on record terminated in convulsions. 5. It is met with in families in which children are subject to head affections or convulsions, but who have also the strumous disposition. 6. It is sometimes met with in connexion with an apoplectic or comatose state from the commencement, as in the cases of crowing apoplexy which I have described. 7. In a great proportion of the cases which terminated fatally there was not the least symptom of head affection through their whole course, if we do not look upon the occasional fits of breathlessness and crowing as indicative of it, and the children were as well, apparently, a few moments before death as they were previous to the first attack of the disease, or as any children could be. 8. The complaint is sometimes, but rarely, attended by cough and permanent difficulty of respiration. 9. Perhaps it may be said that from one-third to half of all the cases of which we have any account, terminated in death. *Dublin Journal of Med. Science. Jan.* 1838.

---

*Observations on the Use of the Stethoscope in the Practice of Midwifery.* By David C. Nagle, A.B., M.B., one of the Physicians to the Dublin General Dispensary.

[This paper does not contain much novelty; but as it states briefly and clearly the chief facts of an important subject and gives some striking illustrations of its practical value, we recommend it to our readers. The following extracts give the more important observations.]

Auscultation affords but two signs of pregnancy: the pulsations of the fœtal heart, and a murmur, that should, correctly speaking, be designated the uterine murmur. The first is absolutely unequivocal; the second equivocal; but, perhaps, the least so of those that should be considered as such. The pulsations of the fœtal heart may be detected, at all events, between the fourth and fifth month, and vary from 120 to 180 in the minute; though I have found them, and that but momentarily, to sink as low as fifty or sixty. They remarkably resemble the ticking of a common watch, and may be detected in various parts of the abdomen, but generally in the iliac regions, particularly during the advanced periods of pregnancy. They are, though occasionally nearly masked by the uterine murmur in the parts, where that is most intense, easily distinguished by an experienced ear, especially if the cylinder be moved a little from the principal site of the murmur; or when the fœtus takes a roll in the womb. They are sometimes liable to be confounded with the pulsations of the mother's heart; but the distinction is drawn with facility by attending to the rhythm, and gradually moving the stethoscope towards the region of the parent's heart. In no case will the beatings of a fœtal heart, when at all energetic, escape detection, if the examination be properly conducted, and the ear of the auscultator be familiar with the rhythm and peculiarity of sound. But this familiarity is absolutely requisite, otherwise the examiner will be left in a state of perplexing uncertainty, where a practised ear would scarcely experience the slightest embarrassment. By an intimate acquaintance with the nature of this sound we can readily ascertain the life or death of the fœtus in the womb; from which knowledge we can derive vastly important practical benefits. We shall also experience no great difficulty in detecting the existence of twins; and, what I have myself found of the utmost, the most gratifying, practical advantage, we can, in most cases, easily discover the nature of the presentation. The other phenomenon, the uterine murmur, is the first of the two stethoscopic signs that occurs; and may, in vigorous constitutions, be detected about the third month. It is not in the least affected by the life or death of the fœtus in the womb; and is quite independent of the fœtal circulation. Its principal site is invariably in the lateral regions, along the course of the lateral uterine arteries; commencing, apparently, opposite the space between the anterior spinous processes, and closely to the iliac arteries. It should not be sought for in the anterior parts of the abdomen; as its occurrence there never happens, unless some branches of the lateral uterine arteries should extend across in an unusually enlarged state. This we can easily verify by moving the stethoscope gradually from the median line towards the ilium; for thus we shall find the murmur to increase in intensity, as we approach the latter. It is frequently detected in both iliac regions at the same time, though usually louder on one side than the other; and by repeating our examination, we shall often hear it on the side, where, in our previous enquiry, we could not, perhaps, discover a vestige of it. To this fact I would beg leave to invite the attention of those, who would persuade us that the placenta must be the seat of this important phenomenon.

Reflection will point out many ways, in which the knowledge, I am endeavouring to inculcate, might be made most usefully available. The following may be considered as no bad instance of it. An intelligent friend of mine was consulted by a female, not long in town, and then appearing to be affected—as she would fain persuade people to think—with some symptoms of dropsy. He suspected the nature of the case, but could get no vaginal examination. He wished me to examine this person, and had a friend of his present, who, I believe, had seen the patient before, but did not consider it a case of pregnancy. We met. It was with difficulty we prevailed on the patient to allow an examination with the stethoscope even. I instantly heard the pulsations of a fœtal heart, and unhesitatingly pronounced it a case of pregnancy. One of the physicians, though long in practice, was sceptical: I wished to remove his uncertainty by getting him to apply his ear to the cylinder. The pulsations of the fœtal heart were very energetic, and about 160 in a minute; whilst those of the mother were remarkably slow, not more than fifty. He could derive no information from the examination, as he was then unacquainted with the use of the instrument. No persuasion could induce the woman to admit the possibility of her being pregnant: but the extreme repugnancc, with which she permitted even the most delicate stethoscopic examination, would be sufficient to create a suspicion that she was not conscious of her own innocence. I requested of my friend to inform her, that I, at least, had not the slightest doubt of her pregnancy; and I even predicted, what actually took place, that she would in a very few days retire into the country for the better concealment of her real state.

The practitioner in midwifery may infer from the case, with which I shall conclude this paper, what important service might be derived from an accurate acquaintance with the use of the stethoscope in that critical department of our profession. We are well aware that infants often appear to be still-born, and are laid by as such, though the labour seemed to proceed quite favorably, and the attendant physician had most carefully discharged his duty. This calamitous result may be occasioned by the pressure exerted on the head at the moment of its escape: or by other circumstances over which the physician could have no control. Auscultation, judiciously employed, would have shewn that, in many such cases, the fœtus had probably enjoyed vigorous existence a few moments previous to its birth; and thus the physician be encouraged to persevere in the use of efficient means for restoring suspended animation. How many valuable lives may be thus preserved! how gratifying to the conscientious physician must success in his professional exertions, under such circumstances, always be! and how simple are the means for the attainment of such encouraging information, may be thus satisfactorily illustrated!

On the 4th of October, 1835, I attended, in her confinement, Mrs. Cooney from Trinity College. The labour proceeded favorably, and the presentation was quite natural. The action of the fœtal heart, examined frequently, as is my custom, was ascertained to be perfectly regular, and retaining its energy undiminished; yet the child, when born, seemed absolutely lifeless; and all appearances, combined with an intolerable fetor of the liquor amnii, which passed off as the head was emerging, were well calculated to leave on one's mind an impression, that the infant was dead for some time. Without the benefit of auscultation I should have been myself under a similar impression; but from the regularity and strength of the heart's action about ten minutes before delivery, when I last explored it, I concluded that the vital spark could scarcely have been quite extinct. No benefit appearing likely to accrue from allowing the cord to remain any longer undivided; and having rapidly provided for the mother's safety, I succeeded, after nearly an hour's unremitting, because not hopeless, perseverance, in restoring the infant to vigorous animation. *Dublin Journal of Med. Science. Jan.* 1838.

---

*Observations on Animal Magnetism.* By Dr. SIGMOND, of London.

[WE give the following narrative without any comment. It will possess greater interest to many of our readers as coming from an eminent physician of our own country. We omit several passages of Dr. Sigmond's paper, and among others his attempt to explain the phenomena on common physiological principles.]

I entered the field of enquiry as a sceptic, and as such, after my enquiries, I remain, as to the belief that any individual is in possession of a power, save that which the strong mind exerts over the weak one, by which he can exercise a preternatural effect over the human frame. I totally disbelieve the existence of any fluid which can, at the will of an operator, be made to pass from his body into that of another, and thus, at his command, produce unwonted sensation.

I merely wish to state what I have observed, and to offer to shew that certain consequences result from a peculiar kind of manipulation, which may easily be acquired, and which, if practised with dexterity, in some instances, might be productive of considerable influence in different conditions of the body. Some weeks since the Baron du Potet de Sennevoy, did me the honour to invite me to be present at a trial of his magnetic power at the University College Hospital. I there saw him perform a series of actions upon different individuals, and he, in two instances, produced what may be termed artificial sleep upon two females, and this was the full extent of his success; his other attempts were failures. The successful cases, however, arrested my attention; they seemed to be the result of simple means, nor could there, at least I thought, be a doubt that the same power existed in any individual who chose to exert it.

I immediately determined to investigate the subject, and for that purpose tried a great number of experiments; but I was most unwilling, for a great length of time, to make my observations at all public, because I thought that I might be accused of seeking notoriety by investigating a subject which rather belonged to the community than to the profession, and one which seemed to be addressed to the popular feeling so easily excited, rather than to the calm and dispassionate consideration of the followers of science. Finding, however, that two distinguished members of the profession, Dr. Elliotson and Mr. Mayo, thought the subject worthy their attention, I persevered in my observations.

The extent of my examination has been such as to satisfy me that I can produce a sleep of a very unusual character, by certain manipulations which do not require me to be in actual contact with the person upon whom the operation is intended to be performed; that I have acquired a certain degree of experience, by which I know how to accommodate the manipulations for the purpose required; and that I can communicate to another individual, in a short space of time, all the information necessary for the production of this sleep.

I commenced my series of experiments by imitating the actions of the Baron Du Potet. My first subjects were of the uneducated class; but I found them so prone to believe in the marvellous—so anxious for extraordinary results, that they deceived both themselves and me. I have since tried the same manipulations upon the higher classes, and though I find them much more sensitive to every impression, and their nervous system more easily acted upon, and although, occasionally, the imagination has led some of them away, yet I have succeeded in giving a very peculiar sleep, amounting almost to stupor, to a vast number of individuals. I have constantly found females much more susceptible of the influence than men; nor does it produce upon them all precisely the same state of sleep. For while in some it is a sort of trance, during which, as often occurs in that unnatural state, pain is scarcely felt, in others it produces hysteria, convulsions, and I have likewise known fainting occur. The most remarkable case that has fallen under my observation, and which, while it excited in me great anxiety, and the deepest interest, has taught me to prosecute my researches with extreme caution, has occurred to me within the last two days. I was enjoying the hospitality of a most amiable family in Fitzroy-square, when animal magnetism became the topic of conversation, and I related the trials I had already made. One of the young ladies proposed to become the subject of experiment, to which I very willingly assented; for, having on former occasions attended her during momentary sickness, I was fully aware of the natural strength of her constitution, and the absence of that nervous temperament which renders this system totally inapplicable. I began what are technically called "the passes." They, as is not unusual, excited laughter and incredulity. I proceeded for about five minutes, and then stopped and enquired if any sensation was produced, and the answer was, "a slight sleepiness;" and ridicule was again thrown upon the subject. I recommenced the manipulations; I observed the eyelids falling, and at last they closed; but, as the same incredulous smile remained, I persevered for three or four minutes, when I, almost doubting whether any influence had been produced, enquired what the feelings were; to this no answer was returned. I found my young friend was in the most complete trance I had ever yet witnessed as the result of my magnetism. The stupor was most profound; and I then tried the usual means to arouse her, but they were vainly exercised. After a few minutes I found the hands become icy cold, the face lost its natural hue, and became perfectly pallid; the extremities became quite cold; the respiration was imperceptible; the stimulus of light did not affect the eye; on speaking to her a faint smile was excited, and a quivering of the lower jaw, which seemed to indicate a wish but an incapability of answering; the pulse became gradually feebler, whilst the external appearance altogether bore such a decidedly deadly cast that naturally some apprehension was excited amongst her family, by whom she was surrounded. Of course I could not but feel a certain degree of anxiety and regret that I had produced such a state, and much uneasiness at the thought that I had inflicted a moment's alarm to my kind friends. These feelings were, however, less acute, from the full knowledge I entertained that the family had long reposed the most perfect confidence in me, and that no member of it had that nervous susceptibility, which would have embarrassed me had any untoward accident presented itself.

I placed the perfectly unconscious subject of this distressing scene in a horizontal position, and directed the application of warmth and of friction to the extremities. Circulation and animal heat were gradually excited, but she presented a most singular appearance of suspended animation. In this condition she remained more than four hours, for I had commenced a little after ten in the evening, and it was about half-past two, that, on some slight effort being made to rouse her, she uttered some of the most piercing shrieks I have ever heard; there were convulsive efforts to raise the limbs; the face, too, became convulsed; she opened her eyes and stared wildly around; she was placed in the upright posture, and seemed sensible. Advantage was taken of this circumstance to carry her to her apartment; before, however, she could reach it, she fell into a profound slumber, but its character was more natural. She was placed in her bed, appearing perfectly composed; the countenance had acquired its natural hue; the respiration was perfectly easy, and the pulse natural. In this state she remained during the whole of the day, until nine o'clock in the evening, once only opening her eyes, and addressing a few words to an anxious and affectionate sister who never left her side. In the evening the young lady joined her family perfectly restored to her wonted cheerfulness. She expressed no complaint whatever. She stated that the feelings that first came over her were those of extreme quiet and repose,—a species of ecstacy,—a gradual languor seemed to steal over her; that she heard something passing around her; felt an inclination, but an utter impossibility, to reply. The first waking-up she, however, described as almost terrific. It was as if she was bursting from a narrow and confined space, and as if she arose from interminable darkness. The lesson that I have thus learnt will not be lost upon me. . . .

I have now exercised this art upon nearly a hundred persons, and with very general success in the fairer part of creation; I have quieted delirium, and given sleep where it has been for many nights vainly solicited. I have magnetised in the presence of many medical men who have been in attendance on the Baron du Potet's lectures, and they have declared that the sleep appears identical with that he produces, and that it is proved by the fact that animals may be sent to sleep by the same movements. I am very anxious that the members of the profession should try the same process. *Lancet. December* 9, 1837.

---

*Practical Observations on Delirium cum Tremore.*
By William Munk, m.d., Lugd. Bat.

Although this paper contains no great novelty it is well worthy the reader's notice. It is always useful to have practical subjects fairly and sensibly discussed; particularly when, as in the case of this disease, there is one line of practice very successful in one class of cases but injurious in another. Its object is to shew "that delirium tremens results from very different causes, that it is frequently complicated with, or even caused by inflammatory affections, that in very many cases the disease is curable by antiphlogistic means only; and that in some of these instances narcotics and stimulants are not only unnecessary but at the same time injurious."

Dr. Munk considers the following statement as an approximation to the truth as to the numerical relation of the different classes of cases. "Delirium tremens," he says, "usually presents itself as a disease of debility, and is benefited by stimulants and narcotics. Next to these, in point of frequency of occurrence, are cases complicated with cerebral inflammation; then follow those cases of the disease of an asthenic nature, but accompanied or complicated with gastric inflammation; and, lowest on the scale, that is to say, of least frequent occurrence, are cases presenting the connexion on which Broussais insists."

Of this form of the disease Dr. Munk relates three cases successfully treated by him; in general terms, he characterizes it as "occurring in individuals of the most abstemious habits, but likewise (and this is the point on which we would particularly insist) in invariably presenting symptoms during life, and appearances after death, which unequivocally denote a well known, because appreciable, organic lesion—that lesion being either gastritis, enteritis, or both conjoined."

These cases were treated by leeches to the epigastrium, mild aperients, and one of them with morphia in addition. With regard to the employment of this remedy Dr. M. observes: "a point in practice may, however, be agitated; we refer to the employment of morphia in these cases. Morphia appears to us injurious when given in intense inflammation of the stomach. The symptoms will be best alleviated by the usual antiphlogistic means—leeching, cold water, ice, &c., but after the violence of the inflammatory symptoms has abated, when it has arrived at the subacute degree, then we know of no remedy possessing equal power with that now under consideration."

*Med. Gazette. February* 10, 1838.

---

*On the Anatomical Structure, and on some of the Morbid Conditions of the Valves of the Aorta.* By T. Watson, m.d.

In a lecture, reported in the Medical Gazette, (vol. xvi. pp. 56, &c.) it was stated by me that the wart-like appearances, seen upon the semilunar valves of the heart, after death, in rheumatic carditis, often form "a kind of double festoon on each valve, from the sesamoid body in the centre to either extremity of its margin, following a natural line of division between the thinner and thicker portions of the valve." The natural structure here referred to, which I believe to be constant, has received but little notice from anatomists. Its relation, however, to a peculiar morbid condition of these valves, makes it worthy of a more particular description.

The cardiac valves consist of a loose duplicature of the delicate membrane that lines the heart; the *endocardium* of modern pathologists. Between its folds is received a thin prolongation of fibrous tissue from the tendinous rings surrounding or constituting the several orifices which are furnished with a valvular apparatus. In the semilunar valves this fibrous substance does not interpose itself between the entire space of the folded membrane. It reaches the free edge of each valve at three points only, namely, at the centre, where it forms the *corpus aurantii,* and at the two extremities. Between these points it stops short, and has a definite limit and outline—a scolloped edge—and so leaves two crescentic portions of the valve, formed merely by the doubled endocardium. In this manner arises the distinction alluded to in my lecture, between the thinner and thicker parts of the valve. The crescentic edges are thin and transparent; the remainder of the valve is more or less thick, firm, and opaque. This structure obtains in the semilunar valves on both sides of the heart, but it is much more conspicuous in some hearts than in others; and more so always in the valves of the aorta, than in those of the pulmonary artery.

It is along the line of union between the scolloped edge of the thicker portion and the inner convex margin of the two thinner crescentic portions, that the minute vegetations (as they have been called) most commonly arrange themselves. Sometimes they follow that double festoon very exactly and completely; sometimes their continuity is broken, and they straggle a little from the line; but still the general tendency to adhere to it is obvious.

*Medical Gazette. December* 16, 1837.

---

*A Selection of Cases presenting aggravated and irregular Forms of Hysteria; and an Analysis of their Phenomena.* No. I. *Hysterical Ischuria.* By THOMAS LAYCOCK, House-Surgeon to the York County Hospital.

THIS paper contains the detail of two cases (one given at great length) of a singular form of disease, which must, some time or other, have engaged the attention and puzzled the judgment of every practitioner of experience. It also contains references to many others of a similar kind, and an abridged account of several. The principal case is that of a young girl, (and all, or almost all, these cases occur in young women,) who, during a period of twelve months or more, presented an infinity of odd, and anomalous, and unaccountable symptoms, comprising, among others, the exhalation of the catamenial and urinary secretion from the umbilicus, mouth, ears, &c. It is remarkable that in this case, as in many others of a similar kind on record, there coexisted manifest imposition on the part of the patient with the real morbid phenomena. On a former occasion (Vol. IV. p. 252,) we referred to this class of diseases, and expressed a wish that the subject were investigated in a philosophical manner. There is none more interesting in the whole records of physic. As contributing some new and authentic materials, Mr. Laycock's paper will be useful: it is highly creditable to his zeal and industry.

*Edinburgh Journal. January,* 1838.

---

*Statistical and Pathological Report of the Royal Infirmary of Edinburgh, for the Years* 1833, 34, 35, 36, *and half of* 1837. By JOHN HOME, M.D.

IT appears from this Report that the managers of the Royal Infirmary, much to their credit, "towards the close of the year 1832, appointed an additional clerk, under the name of Pathological Clerk, whose duty it should be to perform all the post-mortem examinations,—to draw out a particular account of the morbid appearances,—and to insert them in a journal kept for the purpose, called the Register of Dissections, along with a short history of each case, extracted from the daily journals." This office has been filled, since October, 1833, by the author of the present paper, which, although containing nothing very original, is sufficient evidence of his industry and pathological knowledge. The portion of the Report now published is confined to the subject of Phthisis Pulmonalis, and contains a well-digested analysis of the statistical and pathological facts deducible from one hundred fatal cases of this disease. Owing to the numerous classical works that have, of late years, been published on the subject of consumption, more particularly those of Louis and Sir James Clark, the details given in Dr. Home's paper supply us with little that is new: it is, however, an important document and well worthy the attention of the medical statist and pathologist. It is divided into two sections: the first embraces the following circumstances in the history and progress of the disease during life—1, sex; 2, age; 3, occupation; 4, duration of the disease; 5, the influence of season; 6, comparative frequency and mortality; and, 7, peculiarities presented by some of the symptoms. In the second is considered the pathology of the disease, under the following heads:—1, the degree and frequency of the affection of the larynx and trachea; 2, the nature and extent of the affection of the organs of the chest; and, 3, the nature and extent of the affection of the abdominal organs. *Edinburgh Journal. January,* 1838.

---

*Observations relative to Changes in the Crystalline Lens, and the Cause and Cure of Cataract.* By Sir DAVID BREWSTER, K.H. F.R.S.

1. THE method of ascertaining that the change on which presbyopia depends commences generally at the margin of the lens, is to place a taper, or small luminous image, at the distance of thirty or forty feet from the eye. The image of the taper, in place of being perfect, will be elongated in one direction, very frequently downwards, so that the eye is often unable to see the black wick of a candle, in consequence of part of the flame being brought down upon it. If the change were general or symmetrical, a convex lens would remedy the imperfection of vision which it occasions; whereas, it is well known that, until the change has gone round the margin of the lens, the eye does not derive any aid from spectacles. I have especially studied this fact in my own case, and seen it in many other cases.

2. When this change has become symmetrical, and advanced regularly in a sound eye, the application of a lens remedies the imperfection of vision arising from the increase in the focal length of the crystalline. But, if the change has not advanced favorably, either from an improper use of the eye, from general bad health, or from other causes, the fibres, and even the laminæ, *separate;* that is, they cease to be in *optical contact,* in consequence of the lens not being supplied with a sufficiently aqueous secretion. Now, when light passes through a part of the lens where this separation has taken place, it is reflected and decomposed by the fibres, so as to produce not only the prismatic colours, but irregular luminous figures, round the candle. If we now take a plate of brass, with a small hole in it, we may so place it before the eye as to exclude all the light, except that which passes through the diseased part. When this position is found, the eye is *blind:* it can see no objects distinctly, because all the sound part of the lens is shut up. If, on the contrary, we take a small-headed pin, and place the head of it so as to prevent any light from falling on the diseased part of the lens, while the sound part receives rays from any visible object, the vision will be *perfect.* If only a small part of the lens is sound, we may enable a blind eye to see distinctly, by excluding all the light except that which falls on the sound portion of the lens. I have repeatedly performed this experiment, to the surprise of the patient. The colours above referred to are always seen in the crystalline lenses of *animals,* when the laminæ or fibres are separated.

3. The *evidence* that a derangement of fibres arises from mismanagement of the eye when presbyopia commences is not very ample, and has more the character of inference than of demonstration. The separation of the fibres, indicated by coloured images, and irregular luminous branches round and near the principal image, took place in my own eye, and continued for nearly eight months; during which I studied the changes which took place, and made drawings of the luminous figures. As I was in the habit of exposing my eyes to the severest trials, often for twelve and fifteen hours a day, I could not doubt that this *first stage of a cataract* was owing to the greatest mismanagement. It is, besides, universally admitted that, at those periods when the constitution undergoes a change, the greatest attention to the general health is necessary; and, surely, in such a delicate fabric as the crystalline lens, composed of *millions* of fibres, it is highly necessary, when it begins to change its mechanical condition, that it should not be exposed to the same hard work which it might at other times be capable of performing.

4. The experiments by which the seat and extent of the disease may be ascertained have been stated in section 2.

5. I fear it would be presumption in any one, not a medical practitioner, to answer your correspondent's fifth query, respecting the proper remedies for stopping the progress of these changes. I cannot err much, however, in stating what I did in my own case, and leaving your readers to judge whether or not it had any share in the result of restoring my eye to a degree of strength which has enabled it, during the last fifteen years, to perform the hardest work to which that organ can be applied.

Considering the disease as arising from a defect in the secretions by which the lens is supplied with aqueous fluid, I deemed it necessary to give up all experiments,—to abandon reading at night,—to preserve the eye from strong and numerous lights,—to pay the greatest attention to diet,—to take regular exercise; but, above all, to keep clear the primæ viæ, which I did by taking daily the Pulvis salinum compositum for nearly eight months. It is a curious circumstance that I first saw the coloured images, when I was making experiments with my eye, while waiting for the moves of a slow chess-player; and eight months afterwards, in the same room, and similarly employed with the same companion, I all at once observed the images disappear, the fibres and laminæ having then been brought into optical contact. Ever since that time my eyes have been exposed to severe work, and the one which had begun to give way is now the one which I invariably use in all difficult experiments.

6. I believe it is by no means common to see the coloured images that have been above described, but it is extremely common to have the lens so disorganized by inequality of density in different parts, arising, no doubt, from differences in the refractive power of the secretions, that a distant luminous object is not only seen of an irregular form, but is often subdivided into two, or even more, amorphous images. This I have found to be the source of a great many defects of vision which had been ascribed to very different causes.

7. If spectacles are so constructed as to exclude the rays which fall upon the diseased part of the crystalline, the patient will have the advantage of good vision; but, if the sound part is very small, it will be difficult to keep the small aperture opposite to it.

8. As long as the sound part of the lens is of such a magnitude that the patient can see through a small aperture, the art of applying and using which he will acquire by experience, the continued use of the aperture, or the spectacles in which it may be placed, will of course be beneficial.

I have thus answered your correspondent's queries in the way I would have done fifteen years ago; but I have recently been led, by experiments on the crystalline lenses of animals, to more extended views, of which I shall endeavour to give a brief and general idea, referring the reader for more minute information to a paper in the forthcoming volume of the Philosophical Transactions, and to the Reports of the Proceedings of the last two meetings of the British Association.

In these researches I was led to the discovery that the capsule of the crystalline lens is a membrane which performs the functions of endosmose and exosmose, keeping up a due proportion between the aqueous element in the aqueous chamber and the lens. Even in the dead state this membrane imbibes distilled water so greedily that the lens which imbibes it becomes quite soft, expands, and bursts. Viewing the capsule in this light, there can be no doubt that *soft cataract* arises from an excess of aqueous humour imparted to the lens through the capsule; and that *hard* or *dry cataract* arises from a defect of water in the aqueous humour, or an excess of albumen.

Let us now suppose that the skilful oculist has ascertained that there is a disorganization of the lens which does not yet appear as cataract. He will, of course, first try the remedies already mentioned, as they are equally suitable for both varieties; and he may probably succeed in restoring a healthy action in the parts: but, if he finds the disease gaining ground, he must resort to more direct methods before the lens becomes irrecoverably injured.

In this state of matters he should puncture the cornea, so as to get a small portion of the aqueous humour, in order to ascertain, from its refractive power, whether it contains too much or too little albumen. In either case, it might be advisable, by a partial evacuation of the aqueous humour, to reduce the quantity of the diseased secretion, in the hope that a healthier one might be supplied; but, if the disease should continue to advance, distilled water, or a solution of albumen, should be injected into the aqueous chamber, to restore the aqueous humour to its proper condition. If the aqueous humour first taken out should prove to have the ordinary refractive power of that humour, and to appear otherwise in a healthy state, in that case it would not be advisable to introduce either water or albumen. It is quite possible that the capsule of the lens might lose its power of supplying aqueous fluid to the lens, or might supply it too copiously; but such a defect is likely to shew itself either in partial or general opacity. At all events, this opinion will only be entertained in the case where the aqueous humour is found to be in a healthy state.

Although these views are applicable principally when the disease is taken early, yet, even if a lens had *white opacity,* which might not be accompanied with any other disorganization than the mere want of a fluid to restore the optical contact of the fibres, I would not despair of effecting the desired change by the methods above given. *Med. Gazette. Dec.* 30, 1837.

---

### *Case of Pericarditis.* By Thomas Watson, M.D.

[This case is interesting, as shewing the security of the physical diagnostics in the hands of an experienced auscultator. We retain only such parts of the case and of the dissection as bear on the diagnosis.]

On the 11th of October, 1836, I was introduced to a gentleman, thirty-nine years old, with a pale face and sharp thin features, sitting up in bed, breathing shortly and laboriously. His legs were anasarcous, and his belly was tense and fluctuating. I had been previously informed that for years he had been given up to intemperance in drinking, and to indolent and low habits of life. Twice or thrice he had been affected with delirium tremens. He complained to me that he was troubled with wind, shooting upwards through all the left side of his chest: on further enquiry, I found he meant that he had much pain there. A diffused sibilous wheezing was audible in the upper portions of the lungs on both sides: both sides were dull on percussion, in front and behind, at their lower part; and on the right side no sound of respiration could be heard in that part. The jugular veins were swollen and tortuous. His pulse was frequent and very feeble. On applying my ear to the præcordial region, I was immediately sensible of a very loud and harsh *to and fro* sound; a noise of rubbing, apparently close to the ribs, drowning all other sound in that space, keeping time with the alternate motions of the heart, and equally manifest when the patient held his breath. This sound was distinctly perceived by Dr. Sweatman. I stated to him my conviction that our patient was labouring under recent and acute pericarditis. I concluded that he also had hydrothorax; and that, with respect to the heart, there was dilatation of its right cavities at least. I ventured to predict that either Mr. S. would speedily die while the rubbing sound continued, in which case the pericardium would be found inflamed but unadherent, or only partially adherent, to the heart; or, if he survived a few days, the remarkable sound of friction would altogether cease, which would indicate with certainty that adhesion had taken place. . . .

Mercury and some diuretic medicines were prescribed, and leeches were applied to the painful side. I visited Mr. S. again on the 15th. He had been greatly relieved by the treatment adopted, being free from pain and less swelled. The *to and fro* sound, however, continued; but it was less harsh, and confined to a smaller space.

On the 20th I saw him for the third and last time alive. The rubbing noise was entirely gone, and a dull, systolic, deep-seated bellows' sound had taken its place. He was making a vast quantity of urine, the dropsical affection had greatly diminished, and he professed himself to feel much better; but his pulse was scarcely perceptible.

On the 31st it was noticed that he spoke in an odd and unusually loud tone, and his pulse, as Dr. Sweatman informed me, could not be felt; but he said he suffered no pain, and he seemed to breathe with tolerable ease. In the course of that evening he sat up in bed to swallow a dose of medicine; and, having done so, he leaned his head against the nurse, who was supporting him, and presently expired. The body was opened the next day by Mr. Shaw, in the presence of Dr. Sweatman and myself. I did not hesitate to express my certain persuasion that the heart and pericardium were adherent. . . .

There was much fluid in each pleura also. The right lung occupied exclusively the upper part of the cavity, its inferior margin being turned upwards, and fixed by a strong band of adhesion, half an inch in length, to one of the ribs, about midway between the diaphragm and the clavicle; so that the fluid must have been poured out, and have floated the edge of the lung to that height before it adhered. The left pleura was quite healthy. The heart was large. The cavity of the pericardium was entirely obliterated, the opposed serous surfaces of the membrane being united by means of a thick layer of soft mottled lymph: they were easily separated by very gentle traction. All the cavities of the heart were larger than natural: those of the right side were much dilated, the left were filled with dark clotted blood; the walls of the ventricles being considerably thickened. Both the mitral and the aortic valves were morbidly thick and stiff, but there were no "vegetations." *Ibid.*

---

*On the Use of Arsenic in Plethora.* By MR. LAW.

[WE think the following case important, because it affords one instance at least of effectual relief in a most common and, in general, a most untractable disease. Every practitioner of experience will immediately recognize in this case an old acquaintance: we wish we could as surely promise that it exhibits a means of cure likely to be often useful.]

M. F., aged thirty-seven, has for seven or eight years been occasionally subject to violent headach, continuing for some days, and, though not always, very generally proceeding to a hysterical paroxysm, which, along with the headach, is only dispelled by free bleedings of from sixteen to twenty ounces of blood, and sometimes a larger quantity. She is rather above the middle stature, and of a full habit, without much complexion; the bowels apt to be slow, but the necessary aperients regularly employed. She is rather apt to be sedentary, but partakes most sparingly of sleep, or even the recumbent posture, and takes food as well as liquid in much less quantity than the average of her own sex, avoiding vinous stimuli almost entirely. Catamenia regular. Every practitioner summoned to her assistance, wherever she may have been residing, has, from the urgency of the symptoms, been led to the use of the lancet, which alone ever relieved her effectually at the time, the blood flowing with unusual force from the arm when I have had occasion to open a vein in this person. In a few hours after one of these full bleedings, she will leave the recumbent position, proceeding with her usual occupations as if nothing had been the matter. These attacks became of more frequent occurrence towards the end of last spring, when it seemed to me that, as it was next to impossible to diminish the ingesta here, and as the degree of exercise necessary to subdue so strong a disposition to plethora would be of very difficult enforcement, independent of other collateral circumstances in this case, arsenic, administered in small doses at the commencement of an attack, would, from its sedative influence on the system, lessen what we are contending against, and for a longer time than the bleedings, which are so apt to demand repetition. Accordingly, she had a watery mixture prepared, with five minims of the arsenical solution in each teaspoonful, directing her to take a teaspoonful in a little more water, morning and evening, just after a meal, when threatened with an attack, and to intermit it entirely when the tendency disappeared.

It is now six months since the trial was first made, the medicine being, according to these directions, only occasionally employed: nor has she ever since suffered, but in a very slight degree, from what had begun to assume a more alarming aspect. She has on several different occasions in this time found it necessary to have recourse to the arsenic for three successive days, but with the same marked benefit; and, what may appear less accountable, the disposition to such a disease has been thus controlled under even some increase of appetite, using rather more food than formerly. *Edinburgh Journal. January,* 1838.

---

*On the Inhalation of Iodine and Conium in Tubercular Phthisis Pulmonalis.*
By Sir CHARLES SCUDAMORE, M.D.

OUR readers are aware that, several years since, Sir Charles Scudamore published a small work, in which this practice was strongly recommended. Having omitted to notice the exact formula used by him in the cases narrated, he was (we think very properly) found fault with by the critics, and afterwards published the formula which, as amended in his present communication, is as follows:—℞. Iod. gr. viij.; Potassæ Iod. gr. iij.; Alcohol. ℥ss.; Aquæ dist. ℥vss. M. Of this solution, from one drachm to six, and from twenty to thirty-five minims of a saturated tincture of conium are used in each inhalation, which is continued from half an hour to forty minutes. "It is better always to add the conium at the time of using the inhaling. At the temperature of 90°, the volatile properties of iodine are given off very sensibly; but the conium requires more heat, and that of 120° is not too much for the iodine. This degree, therefore, I most recommend; or, if the patient have not a thermometer, let the instruction be to put the water into the inhaler, (first warming it a little to prepare it,) quite as hot as the finger can bear without pain. The inhaler should be kept immersed in rather hotter water during the process. A good glass inhaler also is a material consideration. If it be small, and the tubes too contracted in the bore, the difficulty of inhaling would be great to the invalid, whose respiration is easily embarrassed; whereas, with a fit apparatus, the process is perfectly easy, and not fatiguing." . . . "In the employment of the inhalation, perseverance is necessary, and in some instances for many months. The object sought to be obtained is not merely palliative benefit,—not merely a temporary impression on the morbid function,—but the superseding of the diseased action by a healthy one, and the effecting some organic change."

In the present communication, Sir C. S. professes to give a concise summary of his further experience in phthisis and bronchitis. He refers to cases formerly published by him, and states the favorable result of several of these. The new cases are six, related by Dr. Davidson, including his own case; several of which are much in favour of the practice. Sir C. S. contents himself with stating that he could "relate the cases of a gentleman, aged fifty-four; of a young lady, aged twenty; and of a medical practitioner, aged thirty; in which the most unequivocal symptoms of tubercular disease were strongly developed, in which there was every threatening of danger: and in all of them I was happily quite successful."

It is but justice to Sir C. S. to state that he employs the other ordinary means of treatment together with the inhalation; and our own experience leaves us no room to doubt the great value of the practice as a palliative in phthisis, and as an important remedy in bronchitis. It is true, however, that the relief has often been as great from the simple aqueous as from the medicated inhalation.

*Med. Gazette. February* 17, 1838.

*On Rupture of the Heart, and on Hemorrhage into the Pericardium, without Rupture of the Heart or great Vessels.* By JOHN THURNAM, Esq.

[THIS is an interesting paper, containing three well-detailed cases. We give the last entire, (omitting that part of the dissection not having reference to the subject of the paper,) as it contains a curious explanation of the manner in which blood found in the pericardium may sometimes reach that cavity.]

William Shingleton, aged forty-two, was brought to the Westminster Hospital, December 2, 1837, with very extensive injuries of the bones of the head, ribs, and clavicles: he died almost immediately after being seen by Mr. Bury Dasent, the house-surgeon. It appeared that, during the dense fog which prevailed at the time, he had been knocked down by a runaway horse and chaise. . .

Upon opening the pericardium, it was found to contain a considerable quantity of black fluid blood. Both the right and left cavities of the heart contained semi-fluid black grumous blood, but there was no rupture in any part of this organ, nor in the large vessels contained within the pericardium. In the upper portion of the mediastinum, and reaching to the root of the neck, there was found a considerable layer of coagulated black blood surrounding the bifurcation of the trachea, the œsophagus, superior vena cava, and ascending aorta; and, upon tracing this ecchymosis carefully downwards, it was seen to terminate on the surface of the cava within the pericardium at its highest part, and at the same point there was found to be an irregular laceration of the fibres forming this membrane, through which the blood had evidently gained access into its cavity. Although I did not succeed in demonstrating it, yet I have little doubt that the extravasation had originated in the rupture of some of the large veins in the front of the trachea above the sternum, and that this rupture had been occasioned by the displaced portions of the fractured clavicles. *Ibid.*

